# A Massive Growth in the Palate: A Case Report

**DOI:** 10.7759/cureus.55287

**Published:** 2024-02-29

**Authors:** Manikandan Ramanathan, Sathish Manivel, Sivakumar A, Eileen Mary VC, Logeswari J, Kavitha B

**Affiliations:** 1 Oral and Maxillofacial Surgery, Kauvery Hospital, Chennai, IND; 2 Plastic Surgery, Kauvery Hospital, Chennai, IND; 3 Oral and Maxillofacial Pathology and Oral Microbiology, Meenakshi Ammal Dental College and Hospital, Meenakshi Academy of Higher Education and Research, Chennai, IND

**Keywords:** peripheral ostectomy, mineralizations, periodontal ligament stem cells, palatal growth, peripheral ossifying neoplasms

## Abstract

This case report describes a 67-year-old woman who developed an extensive, slow-growing lesion occupying the whole of the palate in 10 years. Considering clinical and radiographic features, calcifying neoplasms were considered. Correlating microscopic features with clinical features, the lesion was diagnosed as peripheral ossifying fibroma, which seldom presents as an extensive lesion on the palate amongst the elderly age group. This case report will highlight clinicians and pathologists about a rare presentation of a commonly encountered lesion with a comprehensive view of the differential diagnosis of other comparable lesions.

## Introduction

Fibro-osseous lesions refer to a diverse group of lesions affecting the jaws and craniofacial bones, characterized by the replacement of bone by a benign connective tissue matrix exhibiting varying degrees of mineralization [1]. It chiefly comprises developmental lesions (fibrous dysplasia), reactive lesions (cemento-osseous dysplasia), and neoplasms (ossifying fibroma) [2]. Among all, ossifying fibroma and fibrous dysplasia are the two most common lesions to occur. Both result in significant cosmetic and functional disturbances, despite their varied patterns of disease progression [3]. Due to their histological resemblance, these lesions have posed a serious diagnostic dilemma [4].

The most recent 2017 WHO classification of odontogenic tumors assigned “ossifying fibroma” (ICDO from 9262/0 to 9274/0) under fibro-osseous and osteochondromatous lesions [5]. In the jaws, the central type of ossifying fibroma is considered a true neoplasm of odontogenic origin, whereas the peripheral type of ossifying fibroma is a non-neoplastic reactive hyperplastic inflammatory lesion most commonly presenting in the gingiva. Though both entities exhibit distinct biological behavior, both arise from the multipotent mesenchymal cells of the periodontal ligament, which form cementum, bone, and fibrous tissue [6]. Peripheral ossifying fibromas present themselves as small, lobulated gingival masses that measure about 1cm to 1.5 cm in size on average.

Peripheral ossifying fibroma often occurs during the second to fourth decade of life, yet a minority of 0.5% of the cases have been reported in the elderly [7,8]. Sixty percent of these lesions are confined to the anterior maxilla, with sizes ranging from 1 to 1.5 cm. However, large lesions involving the entire palate are sparsely encountered. This paper describes an unusual presentation of peripheral ossifying fibroma of the palate in an elderly female patient for its massive size and rarity and the importance of clinical and radiological correlation in obtaining a definitive diagnosis, along with a review of the literature.

## Case presentation

A female patient age 67 years reported a chief complaint of a large, slow-growing, painless growth in her upper jaw for over 10 years, which was associated with difficulty swallowing and speech. Further inquiry revealed neither a relevant medical history nor a history of deleterious habits. Extra-oral examination revealed no apparent abnormality, and the cervical lymph nodes were not palpable.

**Figure 1:**
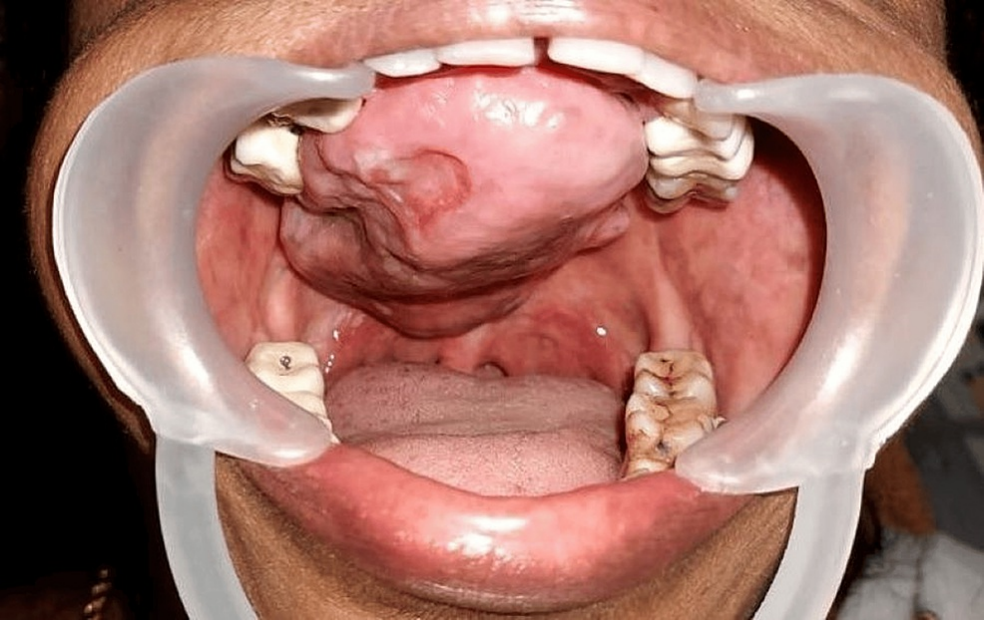
Palatal growth occupying the entire maxilla

On intra-oral examination, an irregular painless growth with a filling effect extending anterior-posteriorly 0.5 cm from the palatal aspect of upper anterior teeth (13-23) to the junction of the hard and soft palate portions, occupying almost the entire length and mediolaterally occupying the entire width of the palate, crossing the midline, and measuring approximately 7x5 x 2.5 cm in size, was present. The lesion was hard in consistency, non-tender, mobile, and exhibited well-defined margins. The surface over the lesion had an indentation imprint of the maxillary molars bilaterally (Figure 1).

**Figure 2:**
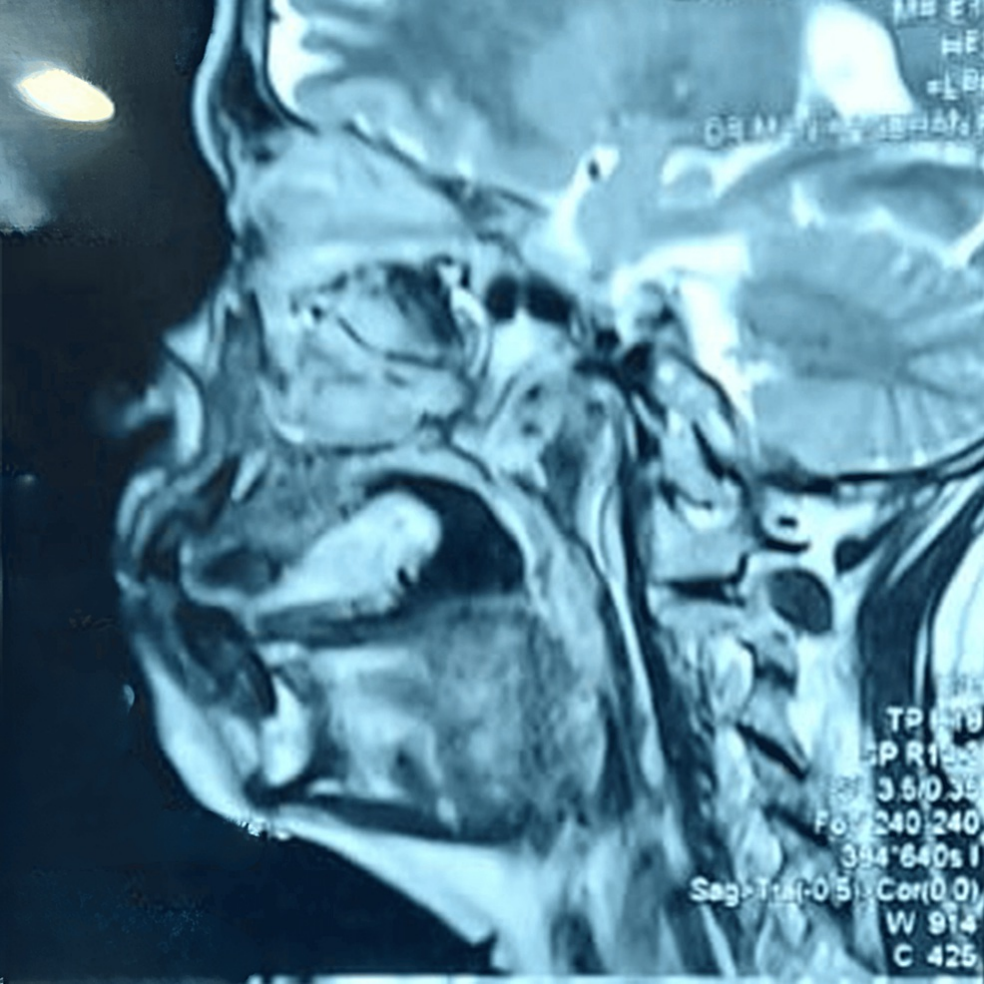
MRI showing sagittal view of the palatal lesion

The MRI and CT analysis of the lesion revealed a faint irregular radiopaque mass in the palate, exhibiting a variable degree of radiodensity, suggesting calcification or mineralization within the lesion. It showed no underlying bone involvement apart from a mild scalloped surface (Figure 2).

**Figure 3:**
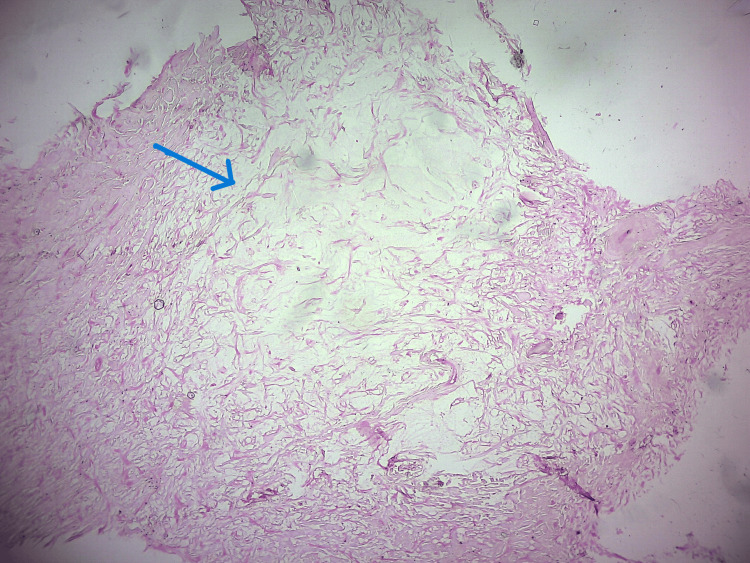
Fibromyxomatous tissue (H&E, 10x)

Based on the above features, the differential diagnoses of fibro-osseous lesions and other calcifying odontogenic neoplasms were considered. An incisional biopsy from the anterior peripheral aspect of the lesion revealed spindle-shaped cells with wavy nuclei in fibro-collagenous connective tissue intermixed with areas of myxoid stroma (Figure 3).

**Figure 4:**
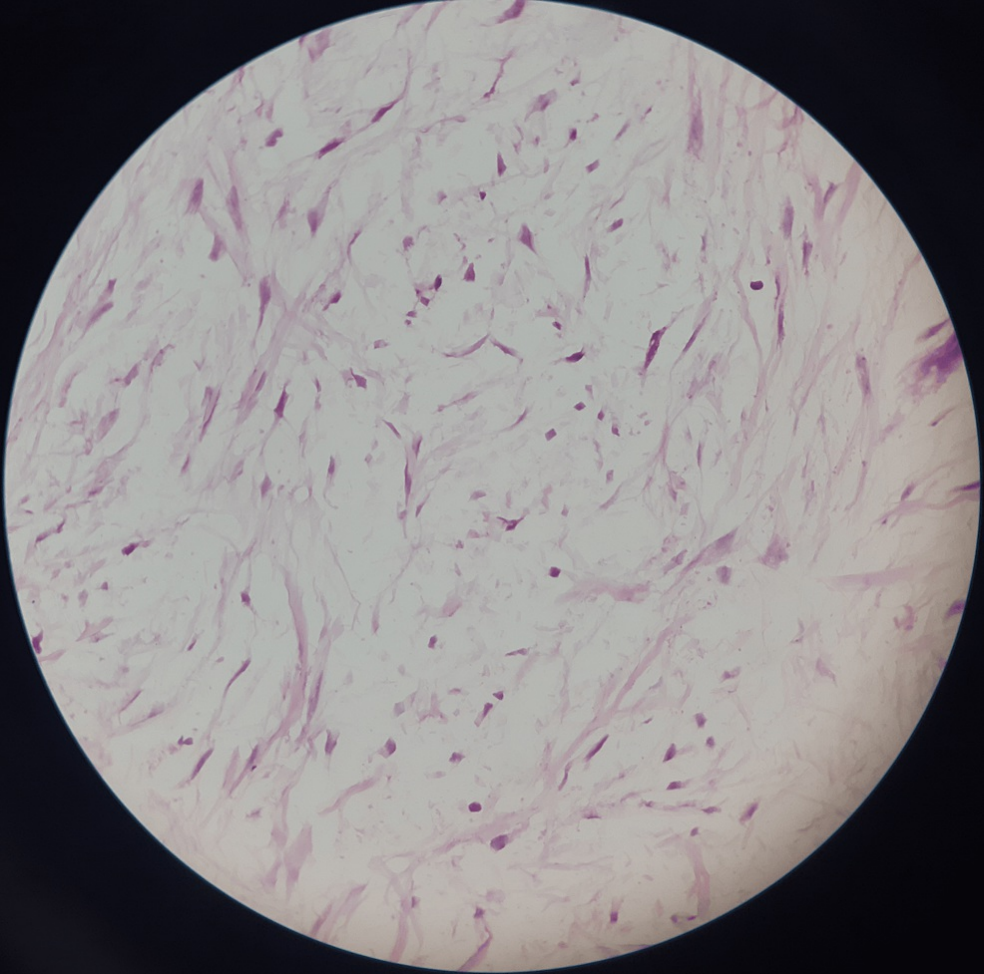
Myxomatous areas (H&E, 20x)

The myxoid stroma was interrupted by thin-walled vessels (Figure 4).

The overlying epithelium was of stratified squamous type. These features were more in favor of fibromyxomatous lesions, and there was no evidence of any hard tissue in the given sample. Since the incisional biopsy was conclusive enough to be consistent with the diagnosis of a benign fibromatous lesion, the presence of calcifications in the radiograph was decided to be assessed upon the excisional biopsy.

Under general anesthesia, the lesion was examined and found to have a rocky consistency. It was pedunculated near the upper right molar area, extending to the buccal aspect. The entire lesion was removed in toto with a 1 cm clearance at the large pedunculated site. Along with the lesion, the premolars (14, 15) and molars (16, 17) were also removed. The lesion was found to have moderate bleeding from the base, and a peripheral osteotomy was done on the entire scalloped underlying area. Since the surgical area was large with bone exposure, a left forearm-free microvascular flap was used to reconstruct the primary defect. Post-operatively, antibiotics and painkillers were advised for seven days.

**Figure 5:**
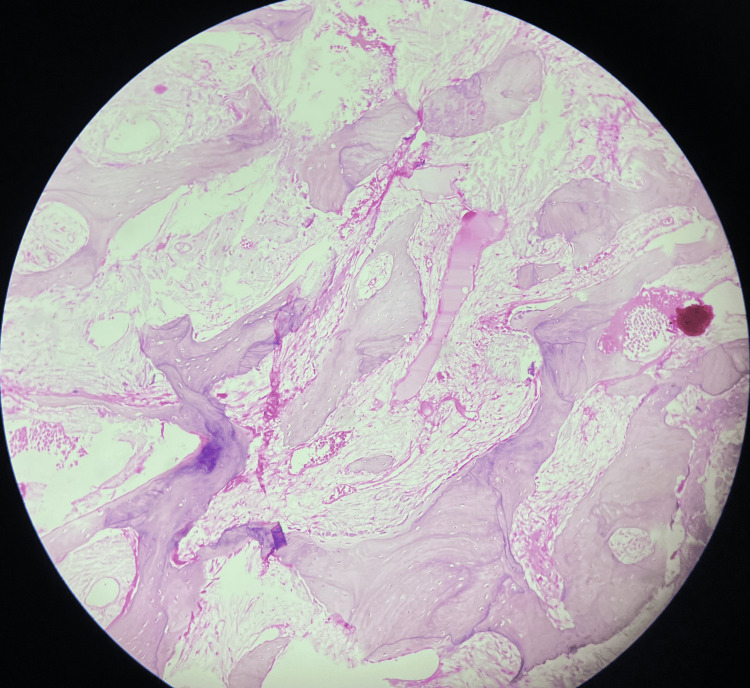
Multiple trabeculae of bones interspersed on the cellular stroma (H&E, 20X)

**Figure 6:**
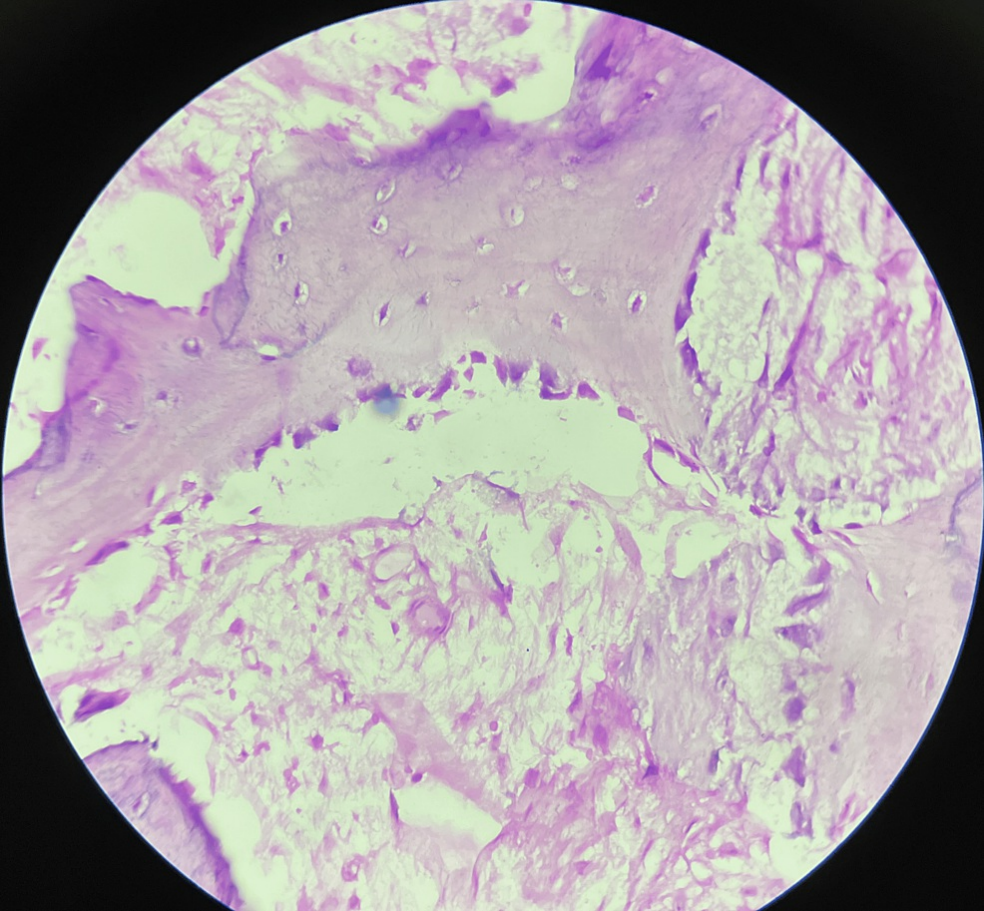
Trabeculae of bone lined by osteoblasts interspersed in cellular stroma (H&E, 40X)

Microscopic examination of the excised lesional tissue revealed fibromyxomatous tissue at the periphery, beneath which numerous interconnecting trabeculae of bone in loose fibrocellular connective tissue were evident (Figure 5). The bony trabeculae exhibited osteoblastic rimming and the presence of osteocytes within the lacunae (Figure 6). Focal areas showed unmineralized osteoid formation. Neither cellular atypia nor increased mitosis were evident.

The differential diagnosis included central ossifying fibroma, peripheral ossifying fibroma, pyogenic granuloma with ossification, peripheral odontogenic fibroma, soft tissue osteoma, and para-osteal osteosarcoma.

The pedunculated appearance of the lesion and the absence of bone involvement helped us rule out the central variant of ossifying fibroma. Pyogenic granuloma often exhibits increased vascularity with proliferating blood vessels in abundance, which was absent in the current case [7]. Though mature lesions with ossifications may be more fibrous, it is rare for pyogenic granuloma to exceed 2 to 2.5 cm in size [8]. On the contrary, our lesion was 7 x 4 x 2.5 cm in size. The absence of odontogenic epithelium and dysplastic dentin in the present case ruled out peripheral odontogenic fibroma, which arises from the odontogenic epithelial cells resting in the periodontal ligament and gingiva [9]. As the lesion was attached to the periosteum, soft tissue osteoma has been ruled out. Though para-osteal osteosarcoma was one of the prime concerns, the lack of cellular atypia, abnormal mitosis, and the absence of bone involvement excluded the diagnosis, thereby deriving the diagnosis of peripheral ossifying fibroma.

## Discussion

The term “ossifying fibroma,” a true benign neoplasm of bone, was designated by Montgomery [10]. The detailed description of ossifying fibroma, published by Sherman and Sternberg in 1948, resolved the long-posed dilemma of considering ossifying fibroma as a separate entity [6]. The term ossifying fibroma was assigned when bone predominates in a lesion, and when psammoma-like calcifications were encountered, the lesion was considered to be cementifying fibroma [6]. Lesions with both bone and cementum-like structures were referred to as “cemento-ossifying fibromas.” Though Eversole’s analysis in 1985 suggested the usage of a common term “ossifying fibroma” for all these lesions since they showed no behavioral or histological difference, the recent WHO (2017) classification considers cemento-ossifying fibroma (ICDO-9274/0) as a distinct type of ossifying fibroma that occurs in the tooth-bearing areas of the jaw and is of odontogenic origin [5,6].

Two categories of ossifying fibroma include the central type and the peripheral type. Central ossifying fibroma is considered a true neoplasm of odontogenic origin. It arises from the endosteum or the periodontal ligament adjacent to the root apex and expands from the medullary cavity of the bone [9]. This lesion appears radiolucent with radiopaque foci capable of causing tooth displacement and root resorption. Cortical bone expansion with a centrifugal growth pattern is a diagnostic feature of central ossifying fibroma.

The peripheral ossifying fibroma is a reactive inflammatory lesion. The occurrence of peripheral ossifying fibroma predominates during the second decade of life and begins to decline beyond the third decade of life. As with an increase in age, there is loss of the periodontal ligament with tooth loss. On the contrary, in our case, the patient was a 67-year-old female. Clinically, peripheral ossifying fibroma occurs on the soft tissue overlying the alveolar process as a smooth, lobulated mass with a pedunculated or sessile base. The incisor-canine region, anterior to the molars, is often involved [7]. The slow-growing nature of the lesion leaves the overlying mucosa intact and remains asymptomatic until a noticeable swelling or deformity results, as in our case, a large intact asymptomatic swelling with a single point of contact in the posterior palate was noticed [6]. Small lesions do not exceed 1.5 cm in diameter, but the literature reports giant lesions of up to 9 cm in size, similar to our lesion, which is 7 cm in size.

According to the literature, the majority of peripheral ossifying fibromas arise from periodontal ligament stem cells. Chronic inflammation causes the differentiation of pluripotent cells into bone cells that are native to that area. However, pertaining to the current case scenario, the location of the lesion in the posterior palate is rare, and this region is devoid of periodontal ligament stem cells, which is unusual for its occurrence.

In vitro studies by Roman et al. [11] and Grimm et al. [12] evidenced multipotent mesenchymal stem cells in the palate similar to the stem cells of the periodontal ligament. The ability of undifferentiated cells to differentiate into any cells of mesenchymal lineage (i.e., osteogenic differentiation) can thus be considered as the probable etiological agent in this case. Any trauma or faulty tissue induction process could be cited as another reason.

Histopathologically, the lesion of interest is a highly fibro-cellular connective tissue intermingled with multiple trabeculae of lamellar bone with an overlying stratified epithelium. Bhaskar and Levin reported the presence of mineralized tissue in 73% of their cases, with bone accounting for 50% and cementum accounting for the remaining cases [13,14]. The histopathological features in typical ulcerated lesions are categorized under three zones as follows [15]: in Zone 1, the superficial zone, fibrinous exudate with polymorphoneutrophils and debris; in Zone 2, beneath the epithelium, proliferating fibroblasts intermixed with chronic inflammatory cells, predominantly lymphocytes and plasma cells; and in Zone 3, the core of the lesion, highly cellular collagenized connective tissue with minimal vascularity. Osteoid and bone formation are evident.

These zones were found to be common for both ulcerated and non-ulcerated lesions, except for the overlying epithelium. In the present case, we were able to observe only Zone 2 in our incisional biopsy, which could be misleading, but the excisional biopsy from the core lesional tissue was consistent with the Zone 3 findings. Further, no absolute histological distinction between bone and cementum existed [15].

The above clinical, radiological, and histopathological features in our case were conclusive in the diagnosis of peripheral ossifying fibroma and did not require other higher investigatory methods. The treatment of choice is complete excision of the lesion, including the periosteum, as done in the current case [14]. Literature shows a recurrence rate of 20% in cases with mineralization [7] and can be expected only in cases of incomplete removal or persistence of triggering factors. The present case of follow-up for two years revealed no recurrence.

## Conclusions

This is a rare case of peripheral ossifying fibroma occurring in the palate, which is an unusual site. Also, the size of the lesion, which was occupying the whole vault of the palate, masqueraded the diagnosis as a common-occurring lesion. This case report will aid clinicians and pathologists in interpreting such massive lesions in the palate.
